# Evaluating the implementation of group empowerment for people with type 2 diabetes at Grassy Park Community Day Centre, Cape Town: A convergent mixed-methods study

**DOI:** 10.4102/safp.v67i1.6192

**Published:** 2025-12-18

**Authors:** Zahraa Saban, Darcelle Schouw, Robert J. Mash

**Affiliations:** 1 Division of Family Medicine and Primary Care, Faculty of Medicine and Health Sciences, Stellenbosch University, Cape Town, South Africa

**Keywords:** type 2 diabetes, group empowerment, health promotion, self-management, self-care, health education

## Abstract

**Background:**

Diabetes is a major contributor to the burden of disease in South Africa, but glycaemic control is poor. Group empowerment and training (GREAT) for people with type 2 diabetes is a cost-effective intervention in our setting. The aim was to evaluate the implementation of GREAT for diabetes at a primary care facility in Cape Town.

**Methods:**

A convergent mixed-method study evaluated implementation outcomes over 6 months. A programme theory model was used to prospectively plan implementation. Semi-structured individual interviews were held with healthcare workers, focus group interviews with patients and sessions were observed to evaluate adoption, feasibility, fidelity and sustainability. Quantitative data evaluated reach, cost and aspects of fidelity. Qualitative data analysis used ATLAS.ti and the framework method. Data were analysed deductively according to the pre-determined implementation outcomes.

**Results:**

Key stakeholders agreed that GREAT was acceptable and appropriate and encouraged adoption. Initial implementation reached 35 patients in four groups and 65% of those invited attended. Only 29% attended all four sessions. Fidelity of the intervention to the training manual was good. Several contextual factors influenced the feasibility of implementation (e.g. support of management, space for group sessions, integration with appointment system, effect on number of walk-in patients, streamlined referral system). Incremental operational costs were trivial (R30/month), while opportunity costs were higher (R26 252/month). Sustainability will be related to ongoing managerial and staff buy-in and adjustment of the implementation strategies to overcome some of the barriers.

**Conclusion:**

The study identified 30 determinants of successful implementation outcomes.

**Contribution:**

Can guide future implementation in similar contexts as GREAT for diabetes is scaled-up in Cape Town and scaled-out to other provinces in South Africa.

## Introduction

Diabetes is a global health problem, and in 2021, there were 537 million adults living with diabetes, which is expected to rise to 783 million by the year 2045.^1^ In 2021, diabetes was responsible for 12.2% of all global deaths in adults.^1^ Global health expenditure on diabetes is expected to reach USD 1.05 trillion by 2045.^1^ In South Africa, diabetes is the second overall cause of death and the first for women.^2^ South Africa has one of the worst records for glycaemic control when compared to similar low- and middle-income countries.^3^ Consequently, diabetes is a substantial burden on patients and families, our health system and our economy.

Most of the population in the Cape Metropole rely on public healthcare. Our primary care facilities serve low socio-economic groups with a lack of education and health literacy.^4^ In the Western Cape, the Practical Approach to Care Kit (PACK) guidelines are used to manage people with diabetes, but glycaemic control remains poor.^5^ The majority of people have uncontrolled diabetes (58% with HbA1c > 8%), and one-third have an HbA1c > 10%.^3^ Good control requires adherence to appropriate treatment and commitment to self-management.^4^ People living with diabetes find self-care challenging because of a lack of knowledge about their condition, poor family support and symptoms such as fatigue, caused by their poorly controlled diabetes or co-morbidities.^5^ This may disempower and demotivate them in terms of self-care and treatment adherence.^5^

Group education for people with type 2 diabetes is effective at improving glycaemic control and is cost-effective.^4,5,6,7,8,9^ The benefit of these structured group programmes is that they allow healthcare professionals to focus their time and energy on consultations and other aspects of diabetes care.^10^ In the Cape Town public sector, the workload and time constraints make it unlikely that comprehensive individual education will take place in the consultation. These group interventions focus on addressing self-care, health literacy, lifestyle changes and treatment adherence while acknowledging the personal, social and economic challenges patients face. Studies that show the effectiveness of group empowerment in diabetic management are mostly from highly resourced settings;^4^ however, the Group Empowerment and Training (GREAT) programme has established its cost-effectiveness in low socio-economic settings in South Africa.^4,9,11^ The implementation of GREAT was previously evaluated in primary care facilities in five provinces in South Africa.^12^

The focus now is on how to implement GREAT for diabetes in various South African primary care contexts and ensure its sustainability and further scale-up. The GREAT programme has never been implemented in the Southern-Western Substructure (SWSS) of Cape Town. The key lessons learnt from previous implementation were considered in this study and were incorporated into a programme theory to guide implementation.^12^ Key lessons included the need for a suitable venue, appropriately trained and sufficient facilitators, support from the whole facility and especially the facility manager, inclusion in clinical governance and health information systems and integration with patient flow and appointment systems.^9^ This study will add further evidence on how to implement GREAT for diabetes in a new substructure with its own contextual issues. The aim was to evaluate the implementation of GREAT for diabetes at Grassy Park Community Day Centre (CDC) in SWSS.

## Research methods and design

### Study design

This study used a convergent mixed methods design to evaluate the following implementation outcomes:

- **Adoption:** To explore why the main stakeholders adopted GREAT for diabetes.
- **Feasibility:** To explore the barriers and enablers to implementation.
- **Fidelity:** To observe the GREAT sessions and to evaluate whether the facilitators delivered the programme as planned and/or had to modify it to make it work.
- **Sustainability:** To explore how well the programme was institutionalised.
- **Reach:** To evaluate how many patients were reached and how many sessions were attended.
- **Cost:** To evaluate the costs involved in implementation.

Qualitative data were collected to explore adoption, feasibility, fidelity and sustainability in the form of semi-structured individual and focus group interviews (FGIs). Quantitative data were collected to evaluate reach, cost and aspects of fidelity.

### Study setting

Grassy Park CDC is in the southern suburbs of Cape Town and serves a community of 82 199 people of mixed ancestry who mainly speak English and Afrikaans.^13,14^ In 2023, data from the Western Cape Single Patient Viewer (SPV) dataset indicated that 56% of patients seen at this facility with diabetes had uncontrolled glucose (HbA1C of > 8%). The CDC did not use a separate ‘club’ system to manage their patients with chronic diseases, and all such patients were seen by appointment within the general practice population. Patients were seen by clinicians, who were either clinical nurse practitioners (CNPs) or doctors. Controlled and uncontrolled patients were seen by both CNPs and doctors and managed according to the PACK guidelines.^15^ Clinicians offered brief health education during the consultation and patients that were referred to the dietician had a 2-month to 3-month waiting period. Diet sheets were given to patients if needed.

### The intervention

Group empowerment and training was a group empowerment programme, training was focused on people with type 2 diabetes.^14^

- **Structure:** Group empowerment and training consisted of four sessions: understanding diabetes, living a healthy lifestyle, understanding medication and preventing complications. Each monthly session was 120 min – 150 min long and conducted by a facilitator. Each session focused on individuals applying new knowledge in their own lives and spent time planning this at the end of each session and giving feedback on how self-management had changed at the start of subsequent sessions. The content was delivered by information exchange using the elicit-provide-elicit model derived from motivational interviewing.^14^
- **Resources:** The facilitator used a variety of resources such as flipcharts and card games to engage the patients. Each facilitator also received a manual that detailed each session and a Diabetes Toolkit that gave background information on diabetes.^16^
- **Communication:** A guiding style of communication was used, which involved collaboration, empathy, evocation, support for autonomy and direction. Facilitators were trained in skills such as the use of open questions, reflective listening and elicit-provide-elicit.

### The implementation strategies

**FIGURE 1:**
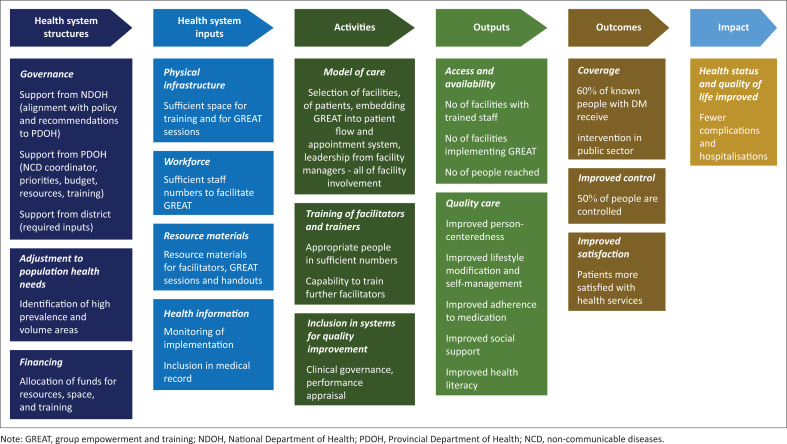
Programme theory for implementation of group empowerment and training.

The study focused on the first 6 months of implementation. The programme theory is shown in Figure 1 and was used to prospectively plan implementation.^12^ This theory model guided the implementation strategies and anticipated contextual factors that could hinder successful implementation.

#### Health system structures

Support was provided by the clinical director of the substructure and facility manager of Grassy Park CDC as well as the local family physician. Grassy Park CDC had a substantial number of patients with type 2 diabetes and could easily identify people needing GREAT for diabetes. No additional financing was made available.

#### Health system inputs

The mosque opposite the facility was used as a venue at no extra cost. The CDC had a multidisciplinary team that was sufficient to implement GREAT with no extra staff members needed. Resource materials were obtained for free from another substructure in collaboration with Stellenbosch University. The implementation of GREAT was monitored by use of a registry for each patient attending each session. Referral to GREAT was documented in the medical record, and attendance was also noted in the folder in the form of a sticker.

#### Activities

Patients with type 2 diabetes, who had a HbA1c > 7%, and those newly diagnosed were referred to the programme. This programme was not intended for people with type 1 diabetes or gestational diabetes. Prior to training, the organisational aspects were discussed using a 10-question planning guide with facility managers and the clinical team responsible for diabetes care.^17^ Various members of the clinical team assisted with identifying suitable patients, drawing folders, providing the medication at the end of sessions and directing patients to the venue. The sessions were delivered monthly, which coincided with the medication collection dates.

Five facilitators (a CNP, a doctor, a nurse, a dietician and a post-basic pharmacy assistant) attended the 3-day training offered by the neighbouring substructure in June 2023 at no extra cost.

Feedback on the implementation of GREAT was given at the clinical governance meetings. The staff members responsible for implementation were encouraged to include their new responsibilities in their performance management by the the facility managers.

### Qualitative data

A descriptive exploratory qualitative approach using semi-structured interviews was followed.

#### Healthcare workers sampling and sample size

This study used purposive sampling to select healthcare workers. The following 10 key informants were selected: (1) the clinical manager of the substructure; (2) the facility manager; (3) two clinicians; (4) the dietician; and (5) facilitators. The final sample size was determined by data saturation when the last two individuals did not identify new issues or potential themes.

#### Healthcare workers’ data collection

Semi-structured individual interviews were conducted with healthcare workers and lasted 30 min - 60 min. The interview guide explored the experience of the intervention, adoption, feasibility, fidelity, opportunity costs and sustainability. The guide was also informed by the Collaboration Framework of Implementation Research and previous interview guides from similar studies.^9,18^

The interviews were conducted 6 months after the implementation of GREAT in the boardroom or consulting room at a mutually convenient time by a research assistant (RA). The interviews were audio-recorded, and field notes were made immediately after. The interviews were conducted in English, the official language of the health services.

#### Patients sampling and sample size

Patients were purposively selected for FGIs. Three groups were interviewed when they completed session four of the programme. Each focus group had between 9 and 12 patients. The final sample size was determined by data saturation when the last focus group did not identify new issues or potential themes.

#### Patients’ data collection

Semi-structured FGIs were used to explore patients’ experiences with the GREAT intervention using an interview guide. The FGIs explored the adoption, feasibility, fidelity, effect and overall patient experience of the programme. The guide was developed using the Collaboration Framework of Implementation Research and a previous study.^9,18^ Interviews were conducted by the RA after the last session and lasted 30 min to 60 min in the same venue. These interviews were audio-recorded, and field notes were made. The interviews were mainly conducted in English with some parts in Afrikaans.

#### Qualitative data analysis

All interviews were transcribed verbatim by a professional transcriber and checked for accuracy by the researcher. The analysis was performed by the first author (ZS), under supervision. ATLAS.ti software assisted with the analysis using the framework method:^19^

- **Familiarisation:** ZS familiarised herself with the transcripts and identified key issues.
- **Development of a coding index:** ZS developed codes from these issues and aligned them deductively according to implementation outcomes.
- **Indexing:** All the data were coded by ZS.
- **Charting:** Coding groups were created according to the implementation outcome categories in step 2 and reports were created that brought all the data together.
- **Interpretation:** The reports were interpreted deductively by ZS in relation to each of the implementation outcomes and themes were identified from the data.

#### Trustworthiness

Data were triangulated from healthcare workers and patients to improve credibility, and respondent validation was conducted with the staff. In terms of reflexivity, ZS was a medical officer at the facility and a novice researcher. She managed people with diabetes at the CDC and initially did not facilitate the sessions. Because of a change in the work profile of one of the facilitators, ZS had to step in and facilitate two out of the four sessions so that implementation could continue. She collected data via an RA to enable openness and honesty in the participants’ responses. Her analysis of the data was informed by her own experience, and her coherence with the data during interpretation was improved by external supervision. The RA held a B.Sc. in Food Science at Stellenbosch University and was a trained health coach. She was not affiliated with the CDC or its staff members.

### Quantitative data

#### Fidelity

Each group of patients was observed once, and each type of session was observed twice. A structured tool was used to observe the GREAT sessions and evaluate fidelity to the content, group facilitation, communication style and skills (Likert scale from 1 rarely, 2 sometimes, 3 mostly, 4 always). This observation tool was validated for use in the original clinical trial^14^ and later adapted for use in the evaluation of GREAT in other provinces.^12^ The RA was a non-participant trained observer, who completed the tool, audio-recorded the sessions and made field notes. The Likert scale was used to calculate mean scores for the various components of the guiding style and communication skills.

#### Reach

Data were collected from the session registers to evaluate the reach in terms of the number referred to GREAT, the number of groups, number of patients that attended and the number of sessions. The GREAT register was cross-checked with the facility’s records to validate the numbers and correct any omissions.

#### Costs

Eligible costs were identified by the researcher with the help of the key informant interviews. Costs included incremental set-up and operational costs to ascertain the monthly and annual costs for the facility. The time spent by staff in implementation was estimated from the interviews and these opportunity costs were calculated by determining the hourly rate per staff category that was provided by the human resources department. Information on unit costs was obtained from both the University and the Department of Health and Wellness.

### Ethical considerations

The study was approved by the Health Research Ethics Committee at Stellenbosch University (S23/06/133), and permission was granted by the Department of Health and Wellness, Western Cape Government.

## Findings

The characteristics of the 10 healthcare workers (HCWs) and 20 patients who participated in the interviews are summarised in Table 1 and Table 2. The themes and subthemes are summarised in Table 3.

**TABLE 1:** Demographics of the healthcare worker interviewees.

Interviewees	Age (years)	Gender	Years of service
Facilitator	34	Female	10
Facilitator	50	Female	27
Facilitator	43	Female	14
Senior clinician	40	Female	14
Senior clinician	43	Female	14
Clinician	55	Female	30
Clinician	57	Female	26
Nurse	45	Female	4
Management	49	Female	25
Management	49	Female	10

**TABLE 2:** Characteristics of patients who participated in the focus group interviews (*N* = 20).

Characteristics	*n*
**Employment status**	
Pensioners	10
Unemployed	5
Employed	3
Unknown	2
**Number of years living with diabetes**	
≤ 6 months	3
> 6 months	17

**TABLE 3:** Themes identified from the health care worker and patient interviews to explore selected implementation outcomes.

Theme number	Theme description
1.	Adoption, acceptability and appropriateness of the intervention
	Burden of disease and need for the intervention
	Alignment with health system goals
	Addressing gaps in management of diabetes
	Manager and staff buy-in
	Perceptions of acceptability and appropriateness
2.	Reach
3.	Feasibility and fidelity
	Coordination and integration of implementation processes
	Patient factors
	Impact on staff, time and material resources
	Session delivery
	Fidelity to the intervention design
	The clinical and social effects of the intervention
4.	Cost
	Incremental and opportunity costs of the intervention
5.	Sustainability of GREAT
	Exploring the sustainability of the intervention

GREAT, group empowerment and training.

### Adoption, acceptability and appropriateness of the intervention

#### Burden of disease and need for the intervention

Many of the respondents were concerned at the increase of people with poorly controlled diabetes. One of the managers highlighted that the poor outcomes were because of a lack of innovation in managing diabetes:

‘I think the diabetic care, that has been the standard of care we have been giving all these many years, has not seen much innovation, there has not been much that we have been doing, the outcomes are pretty much as you would expect.’ (Manager)

Challenges included a high patient load and limited time, combined with stretched human resources and complex patients that needed skilled clinicians. Most providers were nurse practitioners with support from doctors. The volume of patients with complications, multimorbidity and poor glycaemic control were a major cost driver. Several respondents highlighted that if patients were not empowered then poor glycaemic control would continue. All the respondents affirmed the need to implement an empowerment programme that was cost-effective and feasible:

‘I think just the fact that we don’t have a lot of time to spend with patients. There’s a lot that has to be covered in a consultation. You have to do, you know, check blood results, write scripts, check vitals, maybe do a basic examination. By the time you’ve done all of those things, to spend a bit of time talking about diet takes ages.’ (Senior clinician)

#### Alignment with health system goals

A few respondents commented that GREAT was aligned with the facility’s, substructure’s and health department’s goals:

‘So, if you manage diabetes well and you help patients, or even if you prevent it in the first place, but if you, once they are diagnosed, if you help patients manage themselves very well, you prevent that huge cost.’ (Senior clinician)

Many healthcare workers reiterated that this programme would allow patients to take control of their health and make better-informed decisions:

‘But it is easier to get that in your GREAT training. Getting them to understand that this is an ownership that they need to take onto their own and taking care of their health would be their own responsibility and not the doctor’s and the nurse’s responsibility.’ (Facilitator)

Several respondents noted that if patients were better controlled, they could be followed up more in the community and reduce the workload in the facility. A more person-centred community-orientated approach was also aligned with policy goals:

‘If we can have a drop in our chronic patients using the services, which means our footfall will be less. Which means I can [*send*] this patient out to a community service to manage his diabetes. It doesn’t have to come into my facility. And if I can prove all of that, my mother is going to buy it.’ (Manager)

#### Addressing gaps in management of diabetes

Many respondents noted that the lack of time to deliver effective education was one of the main issues that GREAT addressed. Several also mentioned that the group approach allowed more time to deliver education in a non-punitive manner. Some respondents commented that this programme would alleviate pressure on clinicians and fill a gap because of the lack of a health promoter. It was believed that GREAT could make use of the limited resources in an efficient manner:

‘But the fact that they should know it, it’s their illness, it is their body, and they need to take ownership of that. So, it’s very hard to get that across to a patient in a quick 30-minute session.’ (Facilitator)

#### Manager and staff buy-in

The leadership was aware that this programme was implemented successfully at other facilities and commented on the eagerness of clinicians for this programme:

‘I don’t know how much buy-in is at a district level. I know it’s been spoken about like at our forums, which are Metro wide for family physicians. It’s been spoken about, there’s definite enthusiasm among family physicians.’ (Senior clinician)

However, there was some resistance because it was introduced as a research project for one person’s degree and not as an intervention driven by the management. Feedback was mixed on how much support there was among the staff. Some felt, including the managers, that the staff were positive and displayed great interest, while others said they were not well informed or motivated. Some respondents felt that there was management support, while others did not:

‘For projects to be successful at any PHC facility you should have the buy in of the manager because if the manager authorizes, the pharmacy will cooperate, the admin staff will cooperate, the CNP can be released. The doctor can do whatever.’ (Manager)

A few criticised the programme for only focusing on people with diabetes and not their family members. However, most respondents encouraged implementation. The facility manager highlighted positive feedback from patients and was happy that they benefited from the programme.

#### Perceptions of acceptability and appropriateness

Many respondents commented how GREAT was an appropriate intervention that promoted peer support, learning together, shared experiences and allowed participants to ask more questions and gain comprehensive information in a supportive environment. This programme was seen as more acceptable to deliver health education than traditional approaches:

‘Just even in that session, see the power of patients sharing experiences, supporting one another, kind of learning together, and the facilitators offering information and then the patients responding to that and getting the chance to really like think about their lives and their lifestyles and the impact that that has on their health.’ (Senior clinician)

The patients were very positive towards the intervention and highlighted how they found the sessions informative, comprehensive, in-depth and enjoyable. Overall, they found the programme acceptable and appropriate to help them manage their diabetes. They expressed their appreciation for the knowledge they gained and expressed a strong need for earlier intervention:

‘Very informative. And even especially when we got the group here. You felt that you belong.’ (Patient)

### Reach

During the first 6 months, 54 patients were invited and 35 attended GREAT (64.8%) in four groups (Table 4). The majority attended more than half of the sessions although only 28.6% attended all four sessions. Most were pensioners (51.5%) with an average age of 59.5 (s.d. 12.3) years and had type 2 diabetes for more than 6 months (82.9%).

**TABLE 4:** Characteristics of the patients reached.

Characteristics	*n*	%
Number of sessions attended (*N* = 35)		
1	5	14.3
2	9	25.7
3	11	31.4
4	10	28.6
Employment status (*N* = 32)		
Employed	4	11.4
Unemployed	10	28.6
Pensioner	18	51.4
Gender (*N* = 35)		
Female	14	40.0
Male	21	60.0
Number of years living with diabetes (*N* = 34)		
New (≤ 6 months)	5	14.3
Old (> 6 months)	29	82.9

### Feasibility and fidelity of the intervention

**Coordination and integration of implementation processes:** Management highlighted that even with a motivated champion, significant coordination between staff members and entities in the facility was required for implementation to be successful:

‘There is a lot of admin steps going into that to require somebody to coordinate it, who is invested in it. So, you would have to have somebody driving for it to continue.’ (Manager)

All the CNPs and doctors referred patients to the programme. Initially, it was a complex process to refer patients, and this was simplified over time. Even with the streamlining, it took extra time to refer, inform and motivate patients to attend:

‘I don’t think it’s gone that well, to be honest. I think when it comes to knowing what dates are available and trying to get the patients to want to come to GREAT, it’s not been as smooth as it could be.’ (Clinician)

In reception, a designated person was responsible for GREAT, and folders were kept in a special box for the duration of the sessions. The pharmacy played a crucial role in dispensing medication on the day of the session. They advised the clinicians to rewrite all the chronic scripts at the first session so that pre-packed medication would be ready at subsequent sessions. This decreased waiting time and was an incentive for people to attend. Dates for the sessions and for collecting medication needed to be aligned:

‘They were very happy about that fact [*medication incentive*], that they didn’t have to come across the road, because here were quite long waiting times. So, they really appreciated that.’ (Facilitator)

Management highlighted the importance of choosing a suitable and motivated facilitator with good communication skills. There were different views on whether the facilitator should be a clinician. On the one hand, clinicians were more knowledgeable and were able to address some of the participant’s individual clinical needs during or after the sessions. A clinician might have a greater impact on the participants and control of their diabetes. However, taking a clinician away from the queue of patients added pressure on other clinicians and might reduce the number of walk-in patients that could be seen. People debated the benefits of empowering patients versus the negative effects on the queue and consultations that day. Facilitators were put under some pressure to return as quickly as possible to their usual duties:

‘So, the implementation at the facility with the patient wasn’t really the barrier. The barrier was me being in CNP, being out of the facility for those hours of period because it depends on how many clinicians we are in the facility, how many patients has been booked and our un-booked patients.’ (Facilitator)

The venue was a community mosque directly opposite the facility that was previously used to provide services during the coronavirus disease 2019 (COVID-19) pandemic. The venue was free, acceptable and convenient for patients, and the health centre did not have a suitable space. Staff were concerned about being off-site and having to move medication and folders across the road. Security guards helped to deliver medication and direct participants. This choice of venue also aligned with the goal of providing more care in the community:

‘Like I said, we had to shift the human resources, but nothing really. Um, we have a nice relationship with our community. So, the mosque provides us with space. So, I didn’t even have to restructure my facility, but the patients were just there to know that they had to go over to the mosque. And since we have eye clinic there, the mosque is well known …’ (Management)

#### Patient factors

Reasons for declining the programme were lack of awareness, work commitments, transport issues, out-of-area patients, low perceived benefit, acute illnesses, other commitments and family responsibilities:

‘I think for most, the biggest problem is patients, even though a lot of patients are elderly and a lot of them are not working, still seemed to have a lot of commitments when it comes to grandchildren and things like that and doing housework for other people.’ (Clinician)

#### Impact on staff, time and material resources

Two clinicians, one nurse, the dietician and a pharmacy representative attended a 3-day training to be facilitators. Several respondents commented that it would benefit the facility if all suitable staff were trained, as this would allow flexibility for managers in the allocation of staff on the days the programme was conducted. Implementation required a multidisciplinary approach with inputs from clinicians, receptionists, pharmacy and facilitators. This had opportunity costs although the dietician and pharmacy respondent felt that this was part of their usual responsibilities:

‘For us from a practical side, the logistics was more complicated than the implementation. Because remember it’s staff that you’re taking and dedicating to a specific process. So, um, just getting the logistic, we’re very, very few and far between. We’re very limited in our, in our physical resource of staffing.’ (Management)

Although five people were trained, one was rotated away to other duties and could not assist, one often had duties elsewhere and was not always available, one did not fully embody the guiding style and one was not able to deliver all the sessions. It was necessary to train additional facilitators to ensure the sustainability of the initiative. However, no one felt that additional staff should be employed:

‘Consumables you can buy, you can beg, you can borrow, you can steal. Bodies you can’t. People, but motivated people, not just the person. Somebody that actually has a passion for doing it. That is our biggest limitation.’ (Management)

There was some debate about combining the sessions into 2 days, although this would place more cognitive load on the participants and take facilitators away from clinical work for longer. However, it would reduce the number of visits by patients and might improve attendance. GREAT was still seen as an addition to routine care, rather than as part of such care:

‘I’m not sure although that four sessions, if they couldn’t perhaps try to make it easier for the patients by having less sessions and maybe putting more information I know people don’t tend to concentrate for very long but as most of the patients do have to take the whole day to be here anyway, it might not be so difficult for the patient.’ (Clinician)

All the other resource materials were provided during training and so there was no impact on consumables, human resources were the main requirement.

#### Session delivery

Facilitators generally took 2 h - 3 h to organise and deliver a session. The facilitators all thought that the structure and content of the sessions were easy for the staff and patients to understand and relate to. One of the clinicians had some issues with the nutritional section in terms of alternative dietary guidelines, and some patients found the identification of the different food groups challenging. Overall, both patients and facilitators appreciated the guiding style, the content that was delivered and the learning environment that was created:

‘So, the way that the programme has been designed, it’s brilliant.’ (Facilitator)

Different sessions were allocated to different facilitators depending on their background. For example, the dietician conducted the nutrition session, the pharmacy representative conducted the medication session, and the clinicians conducted the sessions on understanding diabetes and complications:

‘You know, because there was really about anatomy and physiology. But it was just for me to get down to the level of my patients. You know, so that they could understand.’ (Facilitator)

The facilitators and patients appreciated the flipcharts and materials used during the sessions, which were relatable and easy to understand. The facilitators found their manual to be comprehensive and easy to understand. The facilitators added fun icebreakers to the sessions to enhance the learning environment:

‘Because when we use the audio visuals it was like, wow is this my body, is that what’s happening, okay so when I eat this is what’s happening, because the illustrations were the keys that was showing that it was insulin, and the teaspoons of sugar were the sugar. Once they understood that they could relate.’ (Facilitator)

Facilitators were flexible when patients missed their sessions and booked them for other groups. However, despite the positive feedback from patients and flexibility of facilitators, there were patients who missed sessions because of administrative errors, family responsibilities, work, weather conditions and acute illnesses. Some patients received medication collection dates that did not coincide with their session dates:

‘Sometimes even if the patient did not come the week before, they do come in and then we don’t turn them away because you were supposed to come last week.’ (Nurse)

#### Fidelity to the intervention design

Eight sessions were observed by the RA, and Table 5 shows the scores for the facilitators’ communication style and skills. Facilitators performed well in adhering to the guiding style and communication skills. The highest scores were for empathy in understanding the perspective and context of participants and for the use of open questions. Facilitators mostly demonstrated respect for participants’ choice and control, evoked ideas and solutions from the group, used active listening and enabled collaboration. The lowest score was for exchanging information using the elicit-provide-elicit model.

**TABLE 5:** Fidelity to the guiding style and communication skills (*N* = 8 sessions).

Communication style and skills	Score
**Guiding style**	
Evocation (mean score 1–4)	3.0
Empathy (mean score 1–4)	3.6
Choice and control (mean score 1–4)	3.4
Collaboration (mean score 1–4)	3.3
Ratio of utterances in guiding style to non-guiding style (ratio)	70:6
**Communication skills**	
Balance of open vs closed questions (mean score 1–4)	3.6
Use of reflective listening and summaries (mean score 1–4)	3.4
Information exchange (mean score 1–4)	2.8

Facilitators adjusted the time needed to deliver the content depending on the group, for example older adults might need longer. In the observed sessions, 80% of the intended content was delivered. Omitting content could be because of time constraints or a lack of preparation:

‘I would sometimes spend a little bit more time or a bit less time on certain topics. So, it depends on what the group also wants, so if they’re asking for a little bit more information on one part I would give them more, they want to spend more time with that particular part, we would spend a lot of time on that, and a bit less with another topic.’ (Facilitator)

### The clinical and social effects of the intervention

A few participants noted significant reductions in their sugar levels, and some mentioned how their symptoms improved. Some noted that they were more conscious of their dietary habits, particularly portion size, incorporating more vegetables in their diet and less sugar-containing foods. They were also more adherent to their medication and increased physical activity. Some of them mentioned how they advocated for a healthier lifestyle among their families and friends. Overall, the intervention empowered the participants to adopt healthier lifestyle behaviours:

‘Ever since I’m here, they educate us how to eat, how to control our sugars and everything. And ever since the day I’m here, I’ve changed my lifestyle, and it’s much better today.’ (Patient)

Managers wanted to document improvement in HbA1c to ensure that the programme was achieving the expected effects. Facilitators observed that some of the patients progressed, and improvements were more evident among those who attended more sessions. One of the senior clinicians indicated that there was already feedback from patients that the programme was effective.

### Cost

**Incremental and opportunity costs of the intervention:** Box 1 summarises the incremental and opportunity costs. During the start-up phase, the incremental costs were R6683 and the opportunity costs R38 561 for the time spent training. The incremental operational costs were trivial at R30/month, and the opportunity costs for the staff time were estimated at R26 252/month or R315 024/year. According to most of the respondents, the short- and long-term benefits of this programme outweighed the opportunity costs. If glycaemic control improved, then patients could attend the facility less often, and there would be a substantial saving on resources and staff time at the facility. Overall, most of the respondents agreed that this implementation was cost-effective:

‘But if anything in a stretched environment this could be a cost-saving initiative and could actually mean we use less resources.’ (Senior clinician)

BOX 1 Incremental and opportunity costs.

**Table:** 

Incremental costs	Amount	Opportunity costs	Amount
**Set-up costs (ZAR)**			
Training venue	0	Staff training	38 561
Resource materials for facility	2773	-	-
Resource materials per facilitator	350	-	-
Refreshments	200	-	-
Reimbursement of trainers	3360	-	-
**Total**	**6683**	**Total**	**38 561**
**Operational costs (ZAR) per month**			
Venue	0	Facilitator time (at 3 h/session)	18 194
Stationary	30	Clinician time (at 5 min/referral)	1763
-	-	Dietician time (at 3 h/session)	2537
-	-	Pharmacy time (at 3 h/session)	3135
-	-	Reception time (at 4 h)	623
**Total monthly cost**	**30**	**Total monthly cost**	**26 252**
**Total annual cost**	**360**	**Total annual cost**	**315 024**

### The sustainability of group empowerment and training

Ongoing commitment to the initiative would depend on confirming the benefits to patients in their HbA1c results. Respondents felt there was a need to monitor the quality and fidelity of the initiative over time and that it would be necessary to train more staff:

‘It would ideal if all clinicians could be trained or most of the, more of the nursing staff if they could be trained on the GREAT training so that if one is not there, someone else can cover them for that particular topic.’ (Facilitator)

There was some debate on the ongoing accessibility and convenience of the community venue. More awareness of the initiative was needed among patients to increase demand and commitment. This could be assisted by community-based services and the patients who attended the sessions. More flexible session times might be needed to improve reach and exploration of the reasons for non-attendance. Messaging services could be used to remind patients:

‘I don’t think they [*patients*] understand what it is that we do at the GREAT sessions, so more education is needed in that instance.’ (Facilitator)

The GREAT programme needed to be integrated into routine care and the logistical and administrative processes streamlined. Ongoing managerial support would be essential and maintaining awareness among the staff. The programme also needed to scale-up and improve its reach. It should be embedded within a comprehensive approach to preventing diabetes and treating people with diabetes within the community and facility. The GREAT programme could also be extended to non-government organisations that run support groups in different venues and offer services via community health workers:

‘Maybe you don’t do it in the facility, you do it off-site at a community centre, or maybe you decide to run shorter more frequent sessions or longer, you know.’ (Senior clinician)

## Discussion

### Summary of the key findings

**FIGURE 2:**
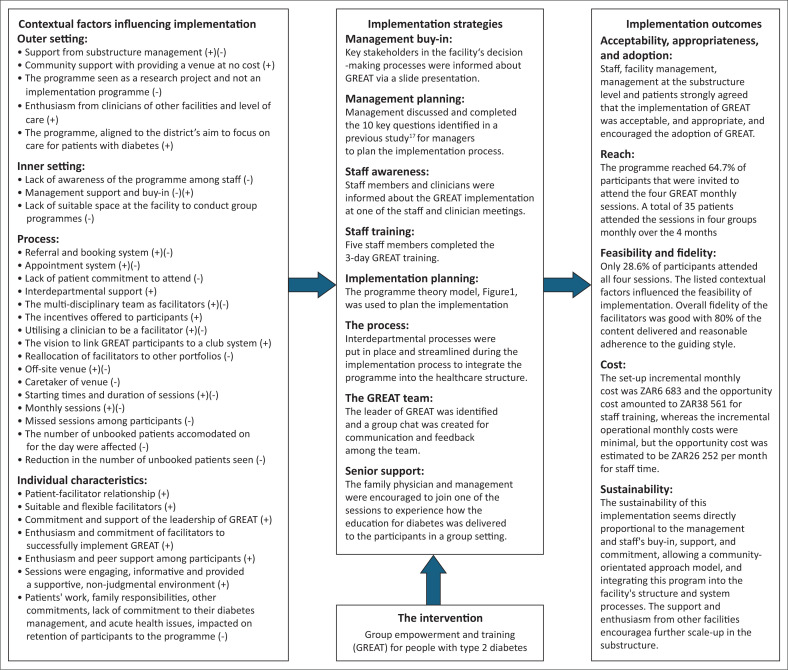
The implementation research logic model.

The key findings are summarised in an Implementation Research Logic Model (IRLM) as shown in Figure 2.^20^ The barriers and enablers to implementation are summarised as contextual factors as well as the extent to which the implementation outcomes were attained. The implementation strategies and intervention itself, as outlined in the methods, are also included to give a complete picture of implementation. The discussion of the key findings focuses on the implementation outcomes and contextual factors.

### Discussion of key findings

The key determinant of implementation was the support and commitment of management at the facility and substructure levels. Even though attempts were made to enable this by informing them timeously, discussing the 10 planning questions for management,^17^ and attaining buy-in, there was still ambivalence. Although the senior leadership of the health system promotes a model of disseminated authority and decision making within a conceptual model of a complex adaptative system, the reality may be different.^21^ The organisational culture is still quite hierarchical and reluctant to innovate without a clear mandate from above.

How do we change the organisational environment? This seems to be key in supporting the implementation of innovations.^17^ Leaders in primary care may need to transform their leadership style so that the organisational culture is more collaborative and open to experiment.^22^ Leadership development is key to transforming and inviting change.^23^ This is a crucial factor to be cognisant of when considering upscaling at other facilities. This could alleviate barriers related to teamwork, logistics, staff allocation and commitment. The management team with the researcher looked at lessons learned from previous studies with the implementation of GREAT and found similar barriers despite trying to mitigate these factors. This suggests that the underlying factor remains the leadership and team dynamics in implementing new programmes in primary care.

The support of the family physician was evident, but they were not actively involved in the implementation process. The role of family physicians and senior clinicians is integral to leadership and governance in the district health system, and utilising their skills and expertise in the implementation of GREAT has been noted elsewhere.^17,24^

The reach of the programme is related to the awareness and understanding of staff members, their motivation to promote it and the number of groups that can be conducted.^15^ The correlation between healthcare worker awareness and the uptake of patients to group empowerment programmes was noted elsewhere.^25^ Clinicians are the preferred choice to refer patients to group diabetes programmes.^25,26^ On average, 65% of referred patients attended the programme, which could be attributed to work commitments, unemployment, family responsibilities, low perceived benefit and other health issues.^25,26^

Several studies indicated that peer support from other patients or community health workers can increase retention, especially among the middle-aged population.^27,28,29^ The positive impact of integrating group diabetes education into community-based services was evident in other studies.^17,27^ Community health workers could be trained as facilitators for people in the community and decrease the burden of utilising health professionals, especially in low-resourced settings. Although community health workers would have less expertise in diabetes and might struggle to address all the questions in the group. In addition, community-based services tend to target the people with better glycaemic control. Similar reasons for poor retention of patients were found in other group programmes for diabetes.^27^ One barrier to patient retention is the misalignment of sessions and medication collection dates.^9^

Suitable facilitators were key to ensure session quality and retention of patients into the programme. The patients who attended the GREAT sessions highlighted how they valued the communication style and skills, the non-judgemental approach and the engaged environment. Using a clinician as a facilitator in this study allowed them to answer complex questions and adjust the prescriptions, if needed, to prevent clinical inertia. This showed a greater impact on glycaemic control, empowering patients with knowledge to elicit self-care and through the regular review of their glucose readings and medication adjustments.

Many facilities do not have space for group education.^17^ The problem was solved by using a community venue, although this created some logistical issues for staff to work outside of the facility. Managers saw the collaboration with the mosque as a positive example of community participation that was aligned with the broader policy on community-orientated primary care.^30,31^ The venue also appeared acceptable to patients, and the use of more accessible community venues can improve retention.^25,32^

This study evaluated implementation in a resource-constrained context, although studies in more developed countries and other ethnic groups observed similar barriers despite having more resources at their disposal.^25,27^ The impact on staff resources and the opportunity costs might be a deterrent for lower-income and resource-constrained settings. In South Africa, in 2009, it was estimated that diabetes caused 2000 amputations annually.^33^ According to a previous study, the estimated mean cost per patient for lower limb amputation in South Africa in 2020 was about $21441.^34^ The total cost per patient that attended the GREAT programme was estimated to be $42 per month or $11 per session and about $72 per person for the entire programme (1$ = ZAR17.9). The GREAT programme therefore is a cost-effective strategy in the South African context,^4,13^ but managers must still decide whether the longer-term benefits warrant the immediate opportunity costs. Better glycaemic control translates into fewer complications and hospitalisations, better quality of life and less workload for the facility in the longer term as patients can be seen less frequently and supported more via community-based services. It may be difficult, however, for managers to take such a long-term view as the opportunity costs are more immediately tangible.

Ongoing management and staff buy-in will depend on seeing the benefits of GREAT reflected in improved HbA1c results. Awareness among patients, a suitable venue and integration of the programme into the model of care are also important. Increased uptake and retention of patients in the programme could be enhanced by peer support from previous participants^27^ and use of electronic reminders.^25^ The effectiveness could be reinforced using digital solutions, such as a WhatsApp Chatbot.^35^ Training additional staff members to facilitate will help spread the load and ensure sustainability. Training could also be reinforced using videos to model and refresh what is expected as well as ongoing monitoring and feedback.

### Strengths and limitations

The mixed-method study design provided a comprehensive evaluation of the implementation outcomes and determinants. This evaluation was strengthened by the triangulation of quantitative and qualitative data, including data from patients and healthcare workers as well as non-participant observations. The IRLM provided a useful structure to make sense of the findings. The detailed description of the context and participants should enable the transferability of the findings to similar contexts in the Western Cape and other parts of South Africa.

The researcher had to facilitate some of the sessions because of facility needs and thus became an embedded researcher. On the one hand, this experience enhanced the interpretation of data, but on the other hand, the researcher had to strengthen her reflexivity to prevent her own experiences unduly influencing the interpretation of data. Interpretation of the data was supervised by an experienced researcher in qualitative research, which mitigated this limitation. Observations were also conducted by an independent RA.

### Recommendations

Key recommendations for the successful implementation of GREAT are:

- Robust management buy-in and support is key to successful implementation.
- Staff awareness about the programme would assist with staff cohesion in implementation and sustainability. One study found that if HCWs observed the sessions, they had a greater belief in the efficacy and improved uptake of patients to the programme.^26^
- Actively utilising the family physicians to provide support to facility managers with implementation.
- Advocacy from peers, reminders of appointments, a suitable venue and staff encouragement to enhance uptake and retention.
- Integrating the programme into the model of care to mitigate some of the logistical issues. More flexible session times may also assist people working or having other commitments to attend or be retained into the programme.
- Investment in leadership development that will promote an organisational culture of innovation and learning will be crucial for future implementation.

Key recommendations on how GREAT could be further implemented or adapted are:

- To target patients who are classified as pre-diabetic and to possibly include family members to enhance a supportive environment at home.
- Group empowerment and training should be extended to other non-communicable diseases in the future.
- To use GREAT within a community-orientated patient care model can be considered for future implementation.
- Managers and planners to ensure new infrastructure can accommodate group activities.
- Short video presentations or session examples can inform and motivate staff to support implementation.
- Broader implications of this implementation suggest that group-based interventions to manage lifestyle diseases are pivotal if barriers are evaluated and mitigated to enhance sustainability. A previous systemic review on guideline implementation highlighted that most implementation programmes focused on education material and empowering patients as implementation strategies, and few focused on assessing barriers to implementation.^36^ Further research could develop strategies to address the barriers to implementation.
- Further research should evaluate how the WhatsApp Chatbot or other digital solutions can be dovetailed with the GREAT programme.

## Conclusion

Group empowerment and training was adopted by all key stakeholders as an intervention that was valued and would decrease the burden of diabetes at the facility. They viewed GREAT as a programme that would have a favourable return on investment. Both staff and patients reported that the programme significantly impacted understanding of diabetes and self-management and provided a supportive and engaging environment. The group dynamics, communication style, incentives, easy access to the programme, utilising suitable facilitators from a multi-disciplinary team, the pivotal roles of management and staff buy-in, were key factors in the success of its implementation. Despite the small number of patients that this programme reached over the initial 6 months, the value of empowering patients and the cost per patient was seen as a justifiable investment and reason to sustain and scale-up the intervention.
